# A Comparative Study to Assess the Efficacy of Two Different Estradiol Formulations during In Vitro Fertilization

**DOI:** 10.1155/2021/3153307

**Published:** 2021-08-10

**Authors:** Manish Banker, Parul Arora, Jwal Banker, Sandeep Shah

**Affiliations:** ^1^Nova IVF Fertility, Ahmedabad, India; ^2^IVI RMA, Madrid, Spain

## Abstract

Improvements in stimulation protocols, introduction of vitrification, and changes in clinical practices have contributed to improved efficacy and safety of assisted reproductive technology (ART) procedures. This has also led to a concomitant increase in number of cycles requiring hormone replacement therapy (HRT) protocol for performing an embryo transfer. Successful implantation is dependent on endometrial thickness which in turn is regulated by temporal regulation of hormones. Careful control of estrogen levels determines uterine receptivity. One of the most used drugs for achieving appropriate endometrial lining of >7 mm in HRT is estradiol valerate. Although different estrogen formulations with varying physicochemical properties exist, there is not enough literature to support if the differences translate into a discernible clinical outcome in an in vitro fertilization (IVF) setting. *Objective and Method*. In this study, retrospective in nature, we compare the efficacy of oral estradiol hemihydrate with estradiol valerate in HRT cycles in 2,529 Indian women, undergoing treatment at a center in India between Jan 2017 and May 2019. *Results*. Our results primarily indicate that between the estradiol valerate and estradiol hemihydrate treatment groups, the implantation rate (IR) was 47.42% and 49.07%, respectively (*P* value 0.284), and the endometrial thickness (mean ± SEM in mm) that was achieved was 9.25 ± 0.038 mm and 9.57 ± 0.058 mm (*P* value < 0.001), respectively. There were no significant differences observed in the secondary outcome measures including clinical pregnancy rate, abortion rate, ectopic pregnancy, and live birth rate. *Conclusions*. Hence, this study concludes that oral estradiol hemihydrate and estradiol valerate are therapeutically equivalent and provide similar clinical outcomes in an IVF setting.

## 1. Introduction

Ever since the first child born in 1978, assisted reproductive technology (ART) has emerged as one of the most significant and successful medical interventions contributing to approximately 0.1% of the global citizens [1]. Faddy et al. in his mathematical modelling projected that there would be approximately 157 million to 357 million people borne through ART by the turn of 21^st^ century [1]. The European IVF-monitoring consortium for the European Society of Human Reproduction and Embryology (ESHRE) report using real-world data from 38 different countries reported that there was a 9.4% increase in the number of ART treatments that accounted for an increase in 7% of child births in 2015 compared to 2014 [2].

The successful cryopreservation of human embryos was first reported in 1983 using slow-cooling technique leading to the first live birth in 1984 [3]. These significant developments changed the course of in vitro fertilization (IVF) treatment and heralded further developments to follow in ART. Eventually, the method of rapid cooling and thawing known as vitrification was introduced which bypassed the difficulties and drawbacks associated with slow cooling. A comprehensive world report on ART for 2004 by the International Committee for Monitoring Assisted Reproductive Technologies (ICMART), noted that the change in clinical practice with reduced number of embryos transferred per attempt yielded favorable outcomes with a considerable decrease in the incidence of multiple births [4]. According to the ICMART 2011 report, the percentage of hormone replacement therapy (HRT) cycles, representing the fraction of frozen-thawed embryo transfer (FET) and oocyte donation cycles to the total embryo transfers, is about 55% [5]. Frozen embryo transfer and blastocyst transfer have been performed more often and have been shown to improve the cumulative pregnancy rates for each patient while eliminating multiple pregnancies [5]. Performance of fresh embryo transfer was seen to decline from 85.5% in 2010 to 79.8% in 2011 with a simultaneous increase in FET from 29.7% in 2010 to 31.3% in 2011 [5]. Frozen embryo transfer has improved neonatal outcomes and seemed to be associated with reduced ectopic pregnancy risk [6]. Earlier studies have illustrated either improvements or no change in ART outcomes following FET [7–12]. Moreover, with the advancements in vitrification techniques, FET provides temporal flexibility and allows the deferred use of all viable embryos obtained from a single egg collection. This has led to the suggestion of a “freeze all” strategy that might facilitate reintroducing the frozen-thawed embryos to a more “physiological” milieu, in harmony with the natural cycle, improving the outcomes [7, 13]. These advancements are quite valuable to those at risk of ovarian hyperstimulation syndrome (OHSS) and those requiring genetic testing to detect chromosomal abnormalities. As a result of such increasing trends due to a change in clinical practice, the percentage of cycles requiring HRT is expected to be higher.

Successful implantation is influenced by the intricate and synchronized crosstalk between good-quality embryos and a receptive endometrium. This initial process is modulated by the spatial-temporal regulation of different hormones at the uterine and ovarian level [14], specifically by estrogen and progesterone. Using a mouse model, a study by Ma et al. [15] has demonstrated that a very narrow range of estrogen levels determines the “window” of uterine receptivity, suggesting the careful regulation of estrogen levels as one of the major factors influencing the outcome in IVF and embryo transfer (IVF-ET) programs.

The regulation of estrogen levels is essential to control uterine receptivity for implantation [15, 16]. Endometrial thickness increases throughout the follicular phase under the influence of estrogen [17]. Following adequate estrogen priming of the endometrium, progesterone prepares the endometrium for implantation [18]. Estrogen enables contraction of spiral arteries resulting in hypoxia in the functional layers and promotes endometrial proliferation. [19]. Since exogenous administration of estrogen is required to increase the serum estrogen within a required range, it is important to be selective and understand the nature of such estrogen interventions. The pharmacological and clinical similarities or dissimilarities need good quality clinical validations.

The common forms in current use are estradiol valerate and micronized estrogens. One of them, estradiol valerate, has been regularly employed in IVF [20, 21]. There is not enough literature to ascertain whether the different esters and salts of estradiol act differently and if any such differences translate into valid clinical significance. Monitoring endometrial thickness in cycles using a HRT protocol provides the best model to study and compare the different estrogen compounds because the endometrium in these cycles is completely under the control of exogenously administered drugs [22, 23]. The aim of our study is to analyzeretrospectively if oral estradiol hemihydrate and estradiol valerate are equally efficacious or dissimilar when used in a HRT protocol. Since we are comparing two different estrogen products, the strongest predictors of estrogenic activity, viz., endometrial thickness and implantation rate (IR), are analyzed as primary outcomes. Secondary outcome measures included in the study are clinical pregnancy rate, abortion rate, live birth rate, and ectopic pregnancy. This study compares the clinical efficacy of two estrogen products in IVF treatment.

## 2. Materials and Methods

This is a retrospective study examining the treatment outcomes of HRT cycles in women during the period from January 2017 to December 2017 (estradiol valerate group) and from October 2018 to May 2019 (estradiol hemihydrate group), at Nova IVF Fertility, Ahmedabad, India. All women (*n* = 2529) who underwent an embryo transfer using the HRT protocol with the transfer of a single/double blastocyst were included in the study. These embryos were either frozen-thawed embryos using self or donor oocytes (OD), or donor embryos (ED), or fresh transfers with embryos using donor oocytes or donor embryos (OD/ED). These samples will be accordingly referred to as thaw self or thaw OD/ED or fresh OD/ED, respectively. Since the study was retrospective in nature, prior ethics approval was not sought. All the participants had consented (written informed) to sharing of their treatment records and outcome before initiating treatment at the clinic, and patient data to produce this paper was used only after their consent for use of their data for research purposes was provided.

### 2.1. Exclusion Criteria

Women suspected or diagnosed to have an endometrial pathology (suspected adhesions, polyps, fibroids, or poor endometrium during HRT) during endometrial preparation or history of poor endometrium were excluded from the study. Women who did not have viable fresh or frozen blastocyst/s for transfer on the embryo transfer day were also excluded.

### 2.2. Study Protocol

The HRT regimen commenced from day 2 of the menstrual period and used estradiol valerate tablets or estradiol hemihydrate 2 mg orally twice daily for 4 days and then 4 mg twice daily for 10 days. Measurement of endometrial thickness was done between the two echogenic borders of endometrium at the midsagittal plane. During the study period Jan 1st–Dec 31st, 2017, all the recruited women were administered estradiol valerate for endometrial preparation. During the study period from 1st October 2018 to 31st May 2019, all women were administered estradiol hemihydrate for endometrial preparation during HRT cycle. Women in either group were assessed on day 10/11 of the menstrual cycle after the initiation of HRT. A transvaginal scan was performed for assessment of endometrial thickness. Adequate endometrial preparation was defined as an endometrial thickness of at least 7 mm with trilaminar appearance [22]. In case the endometrium was not adequate, the estradiol preparation was added vaginally (2 mg twice daily) and the sonography repeated after 4–5 days. Once the endometrium was adequately prepared, serum progesterone levels were measured after the endometrial assessment. Progesterone supplementation was initiated if the serum progesterone values were <0.5 ng/mL. Progesterone was administered orally (dydrogesterone 10 mg twice daily along with micronized progesterone vaginal suppositories 400 mg twice daily). A maximum of two blastocysts were transferred after 5 days of progesterone supplementation. Serum beta human chorionic gonadotropin (B-hCG) levels were measured 14 days after embryo transfer to confirm pregnancy.

### 2.3. Outcome Measures

Outcomes were measured and compared across all HRT cycles using thaw self or thaw OD/ED or fresh OD/ED between the two treatment groups receiving either estradiol valerate or estradiol hemihydrate. Since the objective of the study is to see if there were differences between the two estradiol products, (1) endometrial thickness and (2) IR in HRT cycles were chosen as primary outcome measures. These two measures are directly linked to estrogen activity. Clinical pregnancy rate, abortion rate, ectopic pregnancy rate, and live birth rate were considered as secondary outcome measures.

### 2.4. Statistical Analysis

Statistical analyses of the data were performed using GraphPad Prism 8 software and Microsoft Excel. For the descriptive analyses, results were expressed for numerical data as means ± standard deviations, for categorical variables as number and percentage. The minimum and maximum values are also provided in some cases. The null hypothesis assumed was that the average effect of the two treatments with estradiol valerate or with estradiol hemihydrate is not different. The statistical analysis comparing the data pertaining to primary outcome measures was performed using Welch's *t*-test, and *P* < 0.05 was considered significant. For the secondary outcome measures, statistical analysis using *t*-test for difference in proportions was used and *P* < 0.05 was significant.

## 3. Results and Discussion

### 3.1. Demographic Characteristics

The demographic descriptions of treatment groups are given Tables 1 and 2. There were no significant differences between the two treatment groups.  Table 2 lists the individual demographic variables for the groups classified based on the embryo transfer cycle. There was no significant difference in the variables among these groups.

A total of 3,552 embryo transfers using the HRT protocol, corresponding to 2,476 patients, during the study period were analyzed. The embryo transfer cycle was categorized into three: (a) frozen-thawed transfer using self-oocytes (thaw self), (b) frozen-thawed embryo transfer from OD/ED (thaw OD/ED), or (c) fresh OD/ED transfer cycles (fresh OD/ED). Estrogenic preparation of the endometrium was achieved in all the three embryo transfer cycle categories with forms of estradiol formulations (Table 1). This is a retrospective study, and the two-sample population were assessed for variability. Demographic variables including age, partner age, body mass index (BMI), and years of infertility were comparable between the two treatment groups receiving either estradiol valerate or estradiol hemihydrate (Tables 1 and 2).

### 3.2. Primary Outcome Measures

#### 3.2.1. Endometrial Thickness as a Direct Measure of the Treatment Outcome

The HRT treatment regimen was initiated on day 2 of the menstrual cycle by administering oral estrogen in the form of either estrogen valerate or hemihydrate. In all the treatment groups after estrogen administration, there was an increase in the endometrial thickness (Table 3). Irrespective of the different embryo transfer cycles, the two treatment groups receiving either estradiol hemihydrate or estradiol valerate displayed an adequate endometrial line thickness (Table 3). The endometrial assessment was first performed after 10 days of estrogen initiation. The endometrial line thickness was assessed by transvaginal scan, and it was 9.251 ± 0.038 mm and 9.566 ± 0.0579 mm in the estradiol valerate and hemihydrate groups, respectively (Tables 3 and 4).

#### 3.2.2. Comparison of Implantation Rate

Implantation rate (IR), calculated as the proportion of gestational sacs observed on ultrasonography to the number of transferred embryos, for the two treatment groups is presented in Table 5. IR, expressed as percentage (%), is comparable between the two treatment groups. IR for estradiol valerate versus estradiol hemihydrate in the thaw-self category was 46.52% vs. 47.89%; in the fresh OD/ED category, it was 57.3% vs. 56.7%; and in the thaw OD/ED embryo transfer cycle group, it was 40.98% vs. 44.77%, respectively (Table 5). The statistical analysis of the test of proportions did not indicate any significant difference between the two treatment groups.

### 3.3. Secondary Outcome Measures

The pregnancy outcome was not statistically different between the two groups. Clinical pregnancy rate was comparable between the two groups (58.61% in the estradiol valerate group and 59.57% in the estradiol hemihydrate group). Clinical abortion rate was about 17.86–26.85% in the estradiol valerate-treated group and was about 17.04–22.97% in the estradiol hemihydrate group. Occurrence of ectopic pregnancy events was negligible with only three in the estradiol valerate-treated frozen-thawed embryo transfer cycle and only one observed in the estradiol hemihydrate frozen-thawed using self-oocyte cycle. The occurrence of ectopic pregnancy is nil in the fresh OD/ED or thaw OD/ED groups irrespective of estradiol valerate or estradiol hemihydrate treatment groups. The live birth rate (LBR) was similar between the two groups (Table 6). There was no significant difference in the LBR between estradiol valerate and estradiol hemihydrate treatment groups (45.63% and 47.93%, respectively; relative risk, 0.95; *P* value = 0.532). Comparing the LBR in the two treatment groups, individually for the thaw self or fresh OD/ED or thaw OD/ED categories, it also did not show any significant variation. The rates of biochemical pregnancy (PR), clinical pregnancy (CPR), and clinical abortion (CAB) also did not differ significantly between the two groups (Table 6). Overall, the statistical analysis for difference in proportions did not indicate any difference between the two treatment groups irrespective of the embryo transfer protocols.

## 4. Discussion

The advent of technological advancement, especially vitrification and changes implemented in the practice of ART, has seen significant trend towards increasing number of cycles having an embryo transfer using HRT. This also facilitates genetic testing of embryos, assists those at risk of OHSS, and has a role in fertility preservation [13].

Estrogen supplementation is a crucial and integral part of all HRT protocols. The three common forms of estrogen are estrone (E1), estradiol (E2), and estriol (E3) [15, 24–27]. Estradiol, also referred to as estradiol-17*β*, is the most common and potent form of estrogen [28]. Estrogen could be administered as natural estrogens, conjugated equine estrogen (CEE), or synthetic estrogens. However, estradiol in the natural form has a disadvantage of poor bioavailability [28].

Oral estrogen medications owing to their ease of administration and rapid reversibility are usually preferred in HRT. However, estrogen administered by oral route succumbs to increased rate of metabolism in the gut and liver resulting in higher estrone/estradiol ratio. With a prospect of improving the bioavailability and further enhance the clinical outcomes, alterations of physicochemical characteristics of estradiol have led to the development of varying esters or forms of estradiol. Therefore, esters of estradiol or other synthetic estrogens with chemical variations having potential to offer pharmacological advantages have been regularly employed for the IVF process. Microcrystalline estradiol offers increased surface area and therefore expected to offer greater bioavailability: the smaller the crystal, the better the absorption. Crystals of estradiol hemihydrate contain minimum water and is a repeated stacked arrangement of two molecules of estradiol associated with one molecule of water. Following dissolution, the water molecule does not have a pharmacological role. Estradiol and its hemihydrate form are identical in terms of bioequivalence and activity, with only 3% difference in potency by weight (attributed due to presence of water molecules in hemihydrate form). Estradiol hemihydrate is more hydrated than anhydrous estradiol and is more insoluble in water in comparison, which may result in slower absorption rates with specific formulations of the drug such as vaginal tablets. Administration of 1 mg of oral micronized estradiol resulted in an E2 *C*_max_ of 40–50 pg/mL and an E1 *C*_max_ of 200 pg/mL. Pharmacokinetic parameters with oral administration of 2 mg of micronized estradiol had a *T*_max_ of 8.2 hours and a terminal half-life of 13.5 hours for estradiol. For estrone, *T*_max_ was 6.3 hours and terminal half-life was estimated to be 11.2 hours [29, 30]. Estradiol valerate is an ester of the C17-hydroxy group of estradiol with valeric acid. This formulation prevents the usual metabolism of estradiol to estrone until hydrolysis has taken place. Upon hydrolysis in the intestines to estradiol and valeric acid, the resulting estradiol is rapidly absorbed. During oral treatment, the peak levels of estradiol obtained with either 2 mg estradiol hemihydrate or estradiol valerate were 40 pg/mL on day 1 and 80 pg/mL on day 21 [28, 31]. In terms of indication, AUC (area under the curve), dosing behavior, and adverse effects, there is no difference between both the molecules [32]. Therefore, after oral administration, the pharmacokinetics of estradiol valerate or micronized estradiol remain comparable and the bioavailability is about 5% [30, 33]. Accordingly, estradiol hemihydrate and the valerate are dose equivalent [33]. Although there are clear differences in the physicochemical properties of these formulations of estradiol, whether these translate into a clinically discernible difference is debatable. A recent study comparing the estradiol levels according to dose and formulation in postmenopausal women using hormonal replacement therapy found similar serum estradiol levels with estradiol valerate and hemihydrate [34].

Our study, for the first time, compares two oral formulations of estradiol with varying physicochemical characteristics from the standpoint of clinical outcomes up to live birth rates. Specifically, we analyzed the clinical outcomes in an IVF setting upon exogenous introduction of estrogen in the form of either oral estradiol valerate or estradiol hemihydrate, administered at 2 mg twice daily and then 4 mg. In this study, we have considered only those cycles with the HRT protocol. Our results indicate that treatments with either estradiol valerate or estradiol hemihydrate improved the endometrial receptivity, indicated by increase in endometrial thickness (Tables 3 and 4). The endometrial thickness achieved by both compounds is adequate though there is a significant increase (of 0.351 mm; *P* < 0.0001) in thickness in the hemihydrate group. The cut-off for a thin endometrium has remained variable across different studies, and the clinical outcomes are affected for every millimeter variation in endometrial thickness only below 8 mm for fresh embryo transfer and 7 mm for FET cycles [35]. Although statistically significant, the difference in endometrial thickness between the two treatment groups is minimal (0.351 mm), both above 9 mm, and hence should not translate into any clinically significant difference. The implantation rate measured as the proportion of gestational sacs observed on sonography to the number of embryos transferred did not significantly differ between the two treatment groups (Table 5). These primary results suggest that estradiol hemihydrate is not inferior to estradiol valerate in terms of efficacy. Recent studies by Vartanyan et al. also report increased thickness with estradiol hemihydrate as compared to valerate though it was administered transdermally. They also reported higher implantation rates in the transdermal hemihydrate group [36]. Further, there was no significant difference between the secondary outcomes including clinical pregnancy rate, abortion rate, and ectopic pregnancy. Ultimately, the desired clinical outcome for the success of the treatment is the LBR. There was no difference observed in the LBR outcome of the treatments using these two compounds in all the three subgroups studied. We have not observed any adverse symptoms attributable to overdose of estradiol in any of our study subjects.

## 5. Conclusions

Although it is a retrospective real-life data analysis of the two different preparations of estradiol, this is the first study where the two oral estradiol compounds have been compared for their clinical effects across various treatments in ART involving HRT cycles. The results of this large study (*n* = 2476) show that there is no significant difference between these two different forms of estradiol in terms of efficacy in endometrial preparation (measured as endometrial thickness) and the clinical outcomes (measured as implantation rates, clinical pregnancy rates, abortion rates, and live birth rates) [28, 37].

## Figures and Tables

**Table 1:** Demographic details of the two treatment groups.

	Estradiol valerate	Estradiol hemihydrate	*P* value
*N*	1556	920	
Age (mean ± SD)	33.84 ± 5.44	33.78 ± 5.24	0.6566 (NS)
BMI (mean ± SD)	26.891 ± 5.12	26.63 ± 5.17	0.261 (NS)
Partner age (mean ± SD)	36.56 ± 6.22	36.56 ± 6.40	0.8549 (NS)

NS: not statistically significant; SD: standard deviation; BMI: body mass index.

**Table 2:** Description of patient population in the two treatment groups.

Variable	Estradiol valerate			Estradiol hemihydrate		
	Thaw self	Fresh OD/ED	Thaw OD/ED	Thaw self	Fresh OD/ED	Fresh OD/ED
*N*	734	415	407	453	202	265
Age (years)						
Mean	30.62	36.59	36.82	31.13	36.50	36.27
SD	3.52	5.254	5.27	3.48	4.98	4.646
Min	20	20	23	21	24	23
Max	43	52	50	51	48	49
20–29	265	33	33	162	17	31
30–39	467	260	247	287	125	157
≥40	2	113	130	6	60	78
BMI (kg/m^2^)						
Mean	26.76	27.05	27.01	26.73	26.51	26.54
SD	5.05	5.03	5.34	5.42	4.63	5.14
Partner age (years)						
Mean	33.67	38.88	39.12	34.39	39.09	38.94
SD	4.12	6.45	7.6	4.36	6.48	6.34
Min	23	22	24	23	25	22
Max	67	60	60	52	63	63
20–29	104	14	16	59	8	11
30–39	573	225	195	319	103	135
≥40	56	176	192	53	89	118
Years of infertility (years)						
Mean	4.24	7.53	8.041	4.26	7.045	7.135
SD	3.075	6.503	6.654	3.33	6.079	5.729
Min	0	0	0	0	0	0
Max	18	32	30	17	25	25

OD/ED: donor oocyte/donor embryo; SD: standard deviation; Min: minimum; Max: maximum.

**Table 3:** Endometrial line thickness corresponding to different treatment groups.

	Thaw self		Fresh OD/ED		Thaw OD/ED	
	Estradiol valerate	Estradiol hemihydrate	Estradiol valerate	Estradiol hemihydrate	Estradiol valerate	Estradiol hemihydrate
*N*	734	453	415	202	407	265
Mean thickness (mm)	9.25	9.63	9.324	9.69	9.17	9.37
SEM	0.0592	0.089	0.065	0.114	0.073	0.099
*P* value	0.00047^∗∗^		0.0056^∗^		0.054	

OD/ED: donor oocyte/donor embryo; SEM: standard error mean. ^∗^*P* < 0.01; ^∗∗^*P* < 0.001.

**Table 4:** Endometrial line thickness corresponding to the two treatment groups.

	Endometrial line thickness	
	Estradiol valerate	Estradiol hemihydrate
*N*	1555	920
Mean days	9.251 mm	9.566 mm
SEM	0.038	0.0579
*P* value	*P* < 0.0001^∗∗∗^	

SEM: standard error mean; ^∗∗∗^statistical significance of *P* < 0.0001.

**Table 5:** Implantation rates of the two treatments.

	Total number of embryos transferred	Implantation rate (IR) (%) (ratio)
Thaw self		
Estradiol valerate	1285	46.69% (600/1285)
Estradiol hemihydrate	765	47.89% (365/765)
*P* value	0.646	
Fresh OD/ED		
Estradiol valerate	728	57.3% (402/728)
Estradiol hemihydrate	342	56.7% (197/342)
*P* value	0.4611	
Thaw OD/ED		
Estradiol valerate	718	40.98% (293/718)
Estradiol hemihydrate	452	44.77% (203/452)
*P* value	0.167	
Total		
Estradiol valerate	2731	47.42% (1295/2731)
Estradiol hemihydrate	1559	49.07% (765/1559)
*P* value	0.284	

OD/ED: donor oocyte/donor embryo.

**Table 6:** Measures of secondary outcome of the two treatments.

	Enrolled number of patients *N*	Pregnancy rate (PR) *N*_PR_ (%)	Clinical pregnancy rate (CPR) *N*_CPR_/*N* (%)	Clinical abortion (CAB) *N*_CAB_/*N*_CPR_ (%)	Ectopic (EC) *N*_EC_/*N*_CPR_ (%)	Live birth rate (LBR) *N*_LBR_/*N* (%)
Thaw self						
Estradiol valerate	734	457 (62.26%)	416/734 (56.68%)	91/416 (21.88%)	3/416 (0.72%)	322/734 (43.87%)
Estradiol hemihydrate	453	288 (63.58%)	265/453 (58.50%)	49/265 (18.49%)	1/265 (0.38%)	215/453 (47.46%)
*P* value		0.648	0.535	0.286	—	0.227
Fresh OD/ED						
Estradiol valerate	415	308 (74.21%)	280/415 (67.47%)	50/280 (17.86%)	0	230/415 (55.42%)
Estradiol hemihydrate	202	150 (74.25%)	135/202 (66.83%)	23/135 (17.04%)	0	112/202 (55.45%)
*P* value		0.9912	0.874	0.837	—	0.382
Thaw OD/ED						
Estradiol valerate	407	252 (61.92%)	216/407 (53.07%)	58/216 (26.85%)	0	158/407 (38.82%)
Estradiol hemihydrate	265	167 (63.02%)	148/265 (55.85%)	34/148 (22.97%)	0	114/265 (43.02%)
*P* value		0.773	0.48	0.403	—	0.631
Total						
Estradiol valerate	1556	1017 (65.36%)	912/1556 (58.61%)	199/912 (21.82%)	3/912 (0.33%	710/1556 (45.63%)
Estradiol hemihydrate	920	605 (65.76%)	548/920 (59.57%)	106/548 (19.34%)	1/548 (0.18%)	441/920 (47.93%)
*P* value		0.84	0.625	0.259	—	0.532

PR: pregnancy rate; CPR: clinical pregnancy rate; CAB: clinical abortion; EC: ectopic pregnancy; LBR: live birth rate; OD/ED: donor oocyte/donor embryo; NPR, NCR, NEC, and NLBR: number of instances of pregnancy, clinical pregnancy, ectopic pregnancy, and live birth rate, respectively.

## Data Availability

Data is available on request from the authors. Please contact Dr. Parul Arora (drparul20arora@gmail.com).

## Additional Points

*What is Known*. There has been gradual and sustained increase in assisted reproductive technology (ART) cycles requiring hormone replacement therapy (HRT) due to improvements in stimulation protocols, introduction of vitrification, and changes in clinical practice. Estrogens have been used for successful endometrial preparations in HRT. Due to the major concern with lower bioavailability, several formulations of estrogens have been developed. *What is New*. While there are esters and salt formulations of estrogen with varying physicochemical properties, this large-scale retrospective analysis reviews for the first time to our knowledge any potential clinical advantages of one over the other. Specifically, we have compared for the first time oral estrogen valerate and estrogen hemihydrate in the context of endometrial preparation for various HRT treatment groups in in vitro fertilization (IVF) treatment. We report that these two estradiol preparations are similar in our study.
